# The significance of cysteine synthesis for acclimation to high light conditions

**DOI:** 10.3389/fpls.2014.00776

**Published:** 2015-01-21

**Authors:** Anna Speiser, Stefan Haberland, Mutsumi Watanabe, Markus Wirtz, Karl-Josef Dietz, Kazuki Saito, Rüdiger Hell

**Affiliations:** ^1^Plant Molecular Biology, Centre for Organismal Studies, University of HeidelbergHeidelberg, Germany; ^2^Molecular Plant Physiology, Max Planck Institute of Molecular Plant PhysiologyPotsdam, Germany; ^3^Plant Biochemistry and Physiology, University of BielefeldBielefeld, Germany; ^4^RIKEN Center for Sustainable Resource ScienceYokohama, Japan; ^5^Graduate School of Pharmaceutical Sciences, Chiba UniversityChiba, Japan

**Keywords:** high light stress, SERAT, CYP20-3, chloroplasts, mitochondria, glutathione

## Abstract

Situations of excess light intensity are known to result in the emergence of reactive oxygen species that originate from the electron transport chain in chloroplasts. The redox state of glutathione and its biosynthesis contribute importantly to the plant's response to this stress. In this study we analyzed the significance of cysteine synthesis for long-term acclimation to high light conditions in *Arabidopsis thaliana*. Emphasis was put on the rate-limiting step of cysteine synthesis, the formation of the precursor O-acetylserine (OAS) that is catalyzed by serine acetyltransferase (SERAT). Wild type Arabidopsis plants responded to the high light condition (800 μmol m^−2^ s^−1^ for 10 days) with synthesis of photo-protective anthocyanins, induction of total SERAT activity and elevated glutathione levels when compared to the control condition (100 μmol m^−2^ s^−1^). The role of cysteine synthesis in chloroplasts was probed in mutant plants lacking the chloroplast isoform SERAT2;1 (*serat2;1*) and two knock-out alleles of CYP20-3, a positive interactor of SERAT in the chloroplast. Acclimation to high light resulted in a smaller growth enhancement than wild type in the *serat2;1* and *cyp20-3* mutants, less induction of total SERAT activity and OAS levels but similar cysteine and glutathione concentrations. Expression analysis revealed no increase in mRNA of the chloroplast SERAT2;1 encoding *SERAT2;1* gene but up to 4.4-fold elevated *SERAT2;2* mRNA levels for the mitochondrial SERAT isoform. Thus, lack of chloroplast SERAT2;1 activity or its activation by CYP20-3 prevents the full growth response to high light conditions, but the enhanced demand for glutathione is likely mediated by synthesis of OAS in the mitochondria. In conclusion, cysteine synthesis in the chloroplast is important for performance but is dispensable for survival under long-term exposure to high light and can be partially complemented by cysteine synthesis in mitochondria.

## Introduction

Reactive oxygen species (ROS) play a dual role in plants since they function as regulators and if excessively produced as harmful reactive metabolites. Protein thiols are particularly sensitive to redox regulation, but also to oxidative damage by ROS (Meyer and Hell, 2005). Formation of ROS continuously occurs at low rates during photosynthetic electron transport. However, exposure of plants to high light stimulates release of ROS such as singlet oxygen, superoxide and hydrogen peroxide from the electron transport chain and may cause oxidative stress (Barber and Andersson, 1992; Fryer et al., 2002). The effects on growth of high light and the associated excess excitation energy in chloroplasts have been discussed in literature extensively (Foyer et al., 2009; Suzuki et al., 2012; Szechyńska-Hebda and Karpiński, 2013). Among these responses are morphological changes of the leaves (Eckardt et al., 1997), accumulation of anthocyanins as scavengers of evolving ROS (Chalker-Scott, 1999; Gould et al., 2002; Vanderauwera et al., 2005; Zeng et al., 2010) and the expression of high light-induced genes (Alsharafa et al., 2014). A well-characterized excess light-induced gene is *APX2* that encodes for ascorbate-peroxidase 2, as part of the ascorbate-glutathione ROS detoxification cycle (Rossel et al., 2006; Foyer and Noctor, 2011; Noctor et al., 2011).

Reduced glutathione and its oxidized form represent the most abundant low-molecular weight thiol redox couple found in eukaryotes and prokaryotes and plays a crucial role in adjustment of cellular redox potential and signaling of ROS stress (May et al., 1998; Noctor and Foyer, 1998; Rouhier et al., 2008). Changes of the ratio of reduced to oxidized glutathione and gene expression and activity of glutathione reductase (GR) in response to oxidative stress have frequently been reported (Noctor et al., 2011; Chan et al., 2013). Reduction of oxidized glutathione takes place in the plastids, mitochondria, cytosol and peroxisomes and is so essential for survival of plants that even back-up systems have evolved by NADPH-dependent thioredoxin reductases (Marty et al., 2009). Increases of the steady-state level of the glutathione pool in response to different light conditions are reportedly relatively small (Noctor et al., 2012), but excess oxidized glutathione has been hypothesized to be removed from the cytosolic pool and directed to the vacuole for degradation by γ-glutamyltransferase to recycle cysteine (Grzam et al., 2007; Noctor et al., 2012).

The functional component of glutathione is cysteine that is synthesized from sulfide, the endproduct of the reductive sulfate assimilation pathway in plastids, and from *O*-acetylserine (OAS) by the enzyme *O*-acetylserine (thiol) lyase (OAS-TL). OAS is synthesized from serine and acetyl coenzyme A by serine acetyltransferase (SERAT) whose activity limits the overall rate of cysteine synthesis (Hell and Wirtz, 2011). In *Arabidopsis thaliana* the SERAT protein family is encoded by five isoforms that localize to the cytosol, the mitochondria and the plastids. In unstressed leaves of Arabidopsis the major source of OAS synthesis (79%) are the mitochondria (SERAT2;2) (Haas et al., 2008; Watanabe et al., 2008b), whereas cytosolic SERAT1;1 and chloroplastidic SERAT2;1 contribute 9 and 13%, respectively, of total SERAT activity (Watanabe et al., 2008b). Transcriptional changes in *SERAT* expression levels have several times been observed. Howarth et al. (2003) reported kinetic changes in mRNA abundance of all three major SERAT genes in response to cadmium exposure in leaf and root. *SERAT2;2* and *SERAT2;1* transcripts were increased in roots by treatment with the oxidizing reagent menadione (Lehmann et al., 2009). Transfer of a catalase-deficient mutant (*cat2*) from high CO_2_ concentration to a normal environment resulted in a strong (up to 10-fold) induction of plastidic *SERAT2;1* transcript levels (Queval et al., 2009). Correspondingly, public gene expression databases show up to 15-fold elevations of *SERAT2;1* expression upon H_2_O_2_ treatments (Genevestigator® V3; Hruz et al., 2008; https://genevestigator.com/gv/plant.jsp). With respect to transcription levels and specific activities the cytosolic isoforms SERAT3;1 and SERAT3;2 are considered to be the minor isoforms (reviewed in Hell and Wirtz, 2011).

SERAT activity in the three compartments of cysteine synthesis is modulated *in vivo* by two post-transcriptional mechanisms. In the first mechanism, SERAT and OAS-TL proteins reversibly interact with each other and form the hetero-oligomeric cysteine synthase complex (CSC). Formation and dissociation of the complex constitute a metabolic regulatory model, positioning the CSC as a sensor for cellular sulfur homeostasis (Hell and Wirtz, 2011). Sulfide stabilizes the complex and prevents SERAT from feedback inhibition by cysteine (Wirtz et al., 2012) whereas in the absence of sulfide high OAS concentrations dissociate the complex and thereby adjusting SERAT activity and thus OAS production depending on the actual sulfide supply (Hell and Wirtz, 2011). In the second mechanism SERAT2;1 is postulated to be activated by CYP20-3 mediated CSC association in the stroma (Dominguez-Solis et al., 2008; Park et al., 2013).

Cyclophilins (CYPs) belong to the superfamily of immunophilins, also including FK506- and rapamycin-binding proteins (FKBPs). Both groups of proteins harbor a peptidyl-prolyl *cis/trans* isomerase activity, favoring and accelerating the *cis/trans* transition of peptidyl-prolyl bonds during folding and multimerization of proteins and thus comprising foldase- and chaperone-like functions (Wang and Heitman, 2005). In the Arabidopsis genome 52 immunophilin-encoding genes were identified of which 23 are putative FKBPs and 29 are putative CYP proteins (He et al., 2004; Romano et al., 2004). Subcellular localization studies reveal that 13 CYPs are in the cytosol (Chou and Gasser, 1997), three CYPs are present in the nucleus (Romano et al., 2004), and two CYPs harbor a mitochondrial localization motif. Arabidopsis contains further six genes encoding plastidic CYPs of which only one isoform localizes to the stroma (CYP20-3) (Lippuner et al., 1994), whereas the remaining five CYPs are targeted to the thylakoid lumen. CYP20-3 is the sixth most abundant protein in the stroma (Lippuner et al., 1994; Peltier et al., 2006), suggesting multiple targets for its foldase and chaperone activities. It contains two internal disulfide bonds and its activity was shown to be dependent on thioredoxin-triggered and thus redox-related conformational changes (Motohashi et al., 2003; Laxa et al., 2007).

Investigations of the Arabidopsis *cyp20-3* (= *roc4*; rotamase CYP 4) mutant found a growth retardation under continuous elevated light exposure (up to 1000 μmol m^−2^ s^−1^) and attributed one CYP20-3 function to the repair of photodamaged photosystem II (Cai et al., 2008). Enhanced light intensity (up to 300 μmol m^−2^ s^−1^) and other ROS-inducing conditions (Dominguez-Solis et al., 2008) caused significant growth reduction of the same *cyp20-3* mutant line (Dominguez-Solis et al., 2008). CYP20-3 was shown to interact with SERAT2;1, suggesting some sort of activation or stabilization of the enzyme or the cysteine synthase complex. A mechanism was proposed in which the chloroplast 2-Cys peroxiredoxin may oxidize CYP20-3 and photoreduced thioredoxin then reduces and activates CYP20-3, which in turn would promote cysteine synthesis by its protein-folding capabilities (Dominguez-Solis et al., 2008). This model was extended based on the binding of 12-oxo-phytodienoic acid (OPDA), the precursor of jasmonic acid with independent hormone function, to CYP20-3. Facilitated interaction of CYP20-3 to SERAT2;1 by OPDA was concluded as a key step in hormonal signaling toward cellular redox homeostasis in stress responses (Park et al., 2013).

In this study we investigated whether cysteine synthesis in the chloroplast has a specific role for high light acclimation in Arabidopsis, since it provides the thiol-harboring building block for glutathione. Reasoning that the lack of SERAT2;1 protein in plastids of a null mutant should result in similar or even more severe stress phenotypes compared to CYP20-3 loss of function mutants, we analyzed growth patterns, stress symptoms, cysteine-related metabolites and *SERAT* gene expression. Thus, this study addresses the important question of stress-related redox homeostasis in context of site-specific activity of cysteine synthase complexes.

## Results

### Genomic characterization of *cyp20-3* T-DNA insertion lines

**Figure 1:**
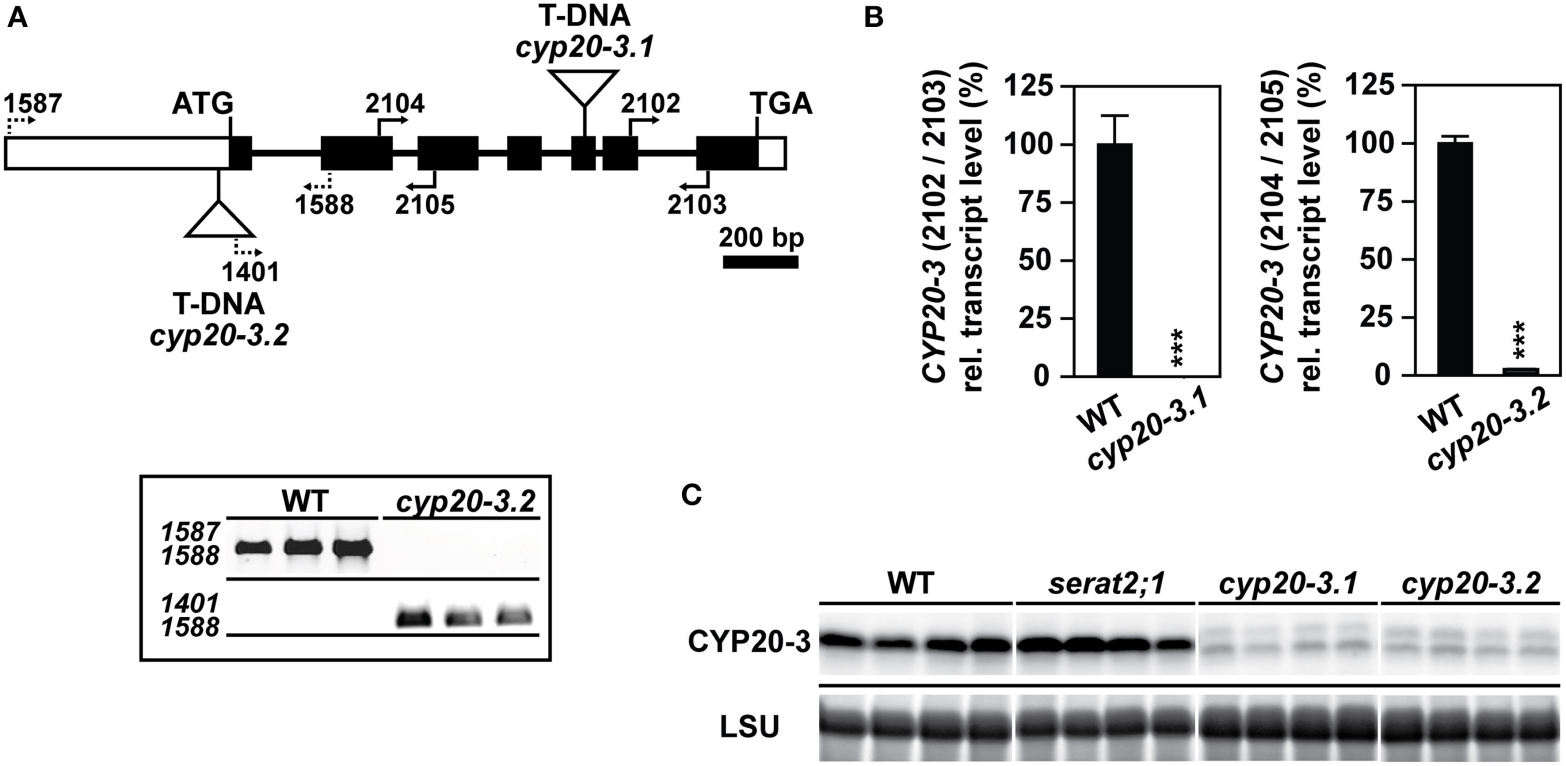
**Identification, molecular characterization and gene expression analysis of *cyp20-3* mutant lines**. **(A)** Gene model of the *Arabidopsis thaliana* gene *CYP20-3* (AT3G62030). Exons and introns are illustrated as black boxes and thin lines, respectively. White boxes represent 5′- and 3′-UTRs. Triangles define the two T-DNA insertions of *cyp20-3.1* and *cyp20-3.2*, respectively. Black arrows and numbering indicate the positions of used primers. Molecular verification of *cyp20-3.2* by PCR using specific primer combinations for the wild type (WT) allele (upper panel) and mutant allele (lower panel). **(B)** *CYP20-3* gene expression levels by qRT-PCR with *cyp20-3.1* and *cyp20-3.2* cDNA and indicated primer combinations. WT transcript levels were set to 100%. Statistically significant differences are indicated as asterisks (^***^p ≤ 0.001; Student's *t*-test). Results represent means ± *SE* (*n* = 3). **(C)** Immunological detection of CYP20-3 using a polyclonal antibody against Arabidopsis CYP20-3 in leaf protein extracts of WT, *serat2;1, cyp20-3.1* and *cyp20-3.2* plants (four lanes each) grown under control conditions. Staining intensities of the large subunit of ribulose-1,5-bisphosphate carboxylase/oxygenase (LSU) protein in the same samples confirm equal loading in the individual lanes.

Two different T-DNA insertion lines of the *CYP20-3* gene as well as one of the *serat2;1* mutant, lacking plastidic SERAT (Watanabe et al., 2008b) were chosen to examine the effects of high light on growth and cysteine synthesis. T-DNA positions and primer binding sites which were used for the molecular characterization of both *cyp20-3* mutants are depicted in Figure 1A. One mutant line (SALK_001615) harbors a T-DNA insertion in the fifth exon and had been previously described as a full knock-out of the expression of the *CYP20-3* gene (Cai et al., 2008; Dominguez-Solis et al., 2008). In these studies this mutant allele was named *cyp20-3* or *roc4* and will herein be referred to as *cyp20-3.1*. Quantitative expression analysis of the *CYP20-3* transcript in *cyp20-3.1* was performed with qRT-PCR (Figure 1B) using 3′ nested primers and demonstrated a minimal expression level of 0.02% of the *CYP20-3* transcript compared to wild type. A so far uncharacterized second T-DNA insertion mutant allele of *CYP20-3, cyp20-3.2* was selected from the SALK-collection (SALK_054125) with the T-DNA residing upstream of the first exon in the 5′-UTR (Figure 1A). Homozygous plants were isolated based on PCR with specified primer combinations (Figure 1A). Transcript analysis with qRT-PCR in homozygous *cyp20-3.2* mutant lines revealed a residual *CYP20-3* expression level of 2.5% compared to wild type plants (Figure 1B). A polyclonal antibody against Arabidopsis CYP20-3 detected residual amounts of CYP20-3 in *cyp20-3.1* and *cyp20-3.2* that corresponded to the detected remaining mRNA levels (Figure 1C). It should be noted that no CYP20-3 protein was detected in *cyp20-3.1* using a different antibody in an earlier study (Cai et al., 2008). Thus, both alleles are strong knock-down mutants of the *cyp20-3* gene. The CYP20-3 protein level was unchanged in the *serat2;1* mutant compared to wild type, excluding any compensatory upregulation in response to the lack of this interaction target.

### Acclimation to long-term exposure to high light conditions

**Figure 2:**
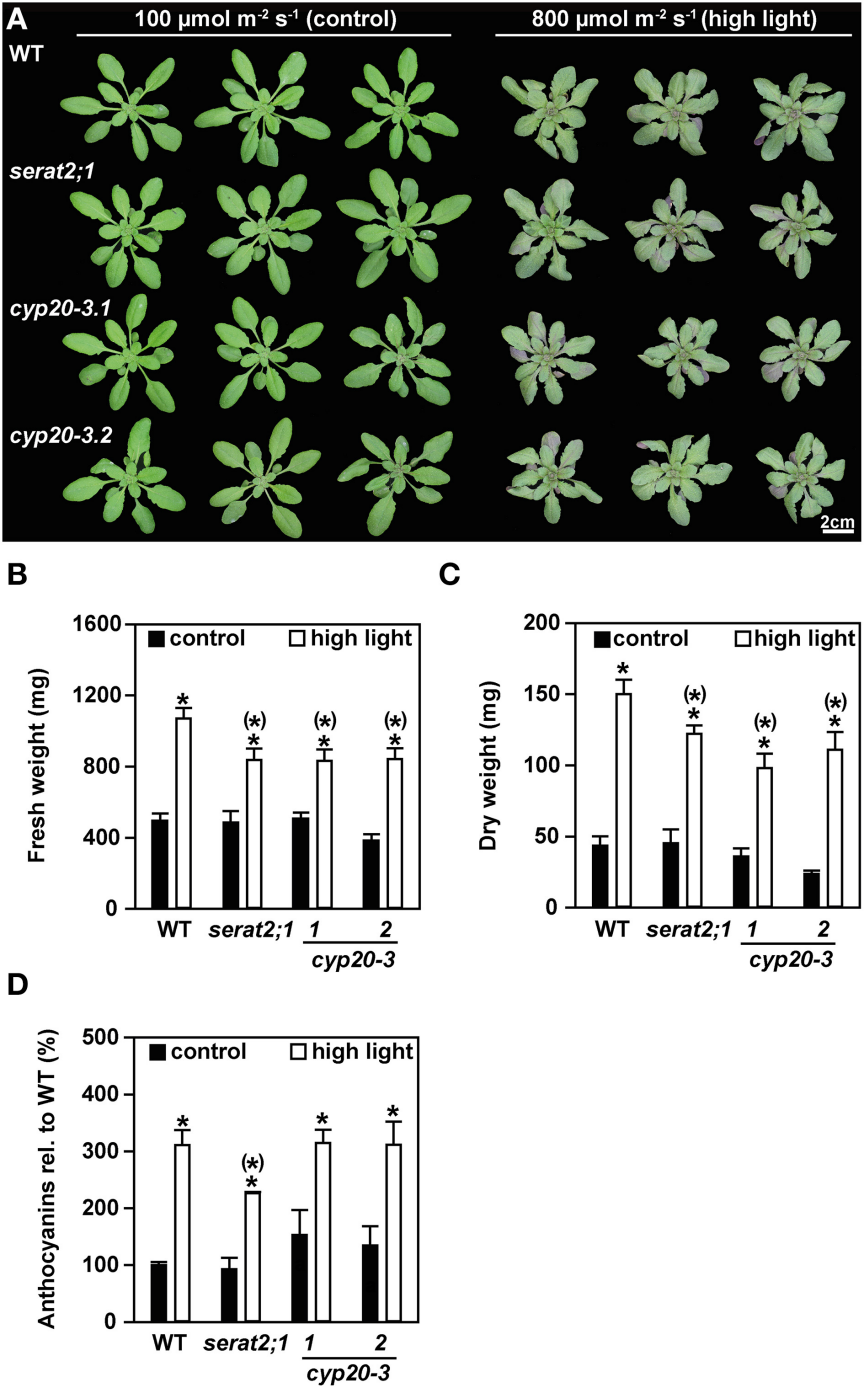
**Effect of high light (800 μmol m^−2^ s^−1^, 10 days) on the phenotype, fresh weight and anthocyanin content in wild type (WT) and *serat2;1, cyp20-3.1*, and *cyp20-3.2* mutant lines**. **(A)** Phenotype of three representative WT and mutant plants, respectively, that were subjected to control conditions of 100 μmol m^−2^ s^−1^ (long-day) and high light conditions of 800 μmol m^−2^ s^−1^ (long-day) for 10 days after 3 weeks of growth under short-day and low light conditions. **(B)** Fresh weight analysis of whole rosettes from control and high light-treated plants. Bars denote data means of 8–10 sampled individuals. **(C)** Dry weight determination of 3–5 sampled individuals from control and high light-treated plants. **(D)** Measurement of anthocyanin contents in control and high light-treated plant leaves. WT anthocyanin levels were set to 100% (0.2 μg mg^−1^ FW). Results represent means ± *SE* (*n* = 3). Black bars indicate control conditions, whereas white bars outline high light treatment. Asterisks indicate statistically significant differences between control and high light treatment (*p* ≤ 0.05; Student's *t*-test). Asterisks with parentheses point to the comparison with high light treated WT (*p* ≤ 0.05; Student's *t*-test). FW, fresh weight.

The effect of high light on Arabidopsis mutants with eliminated plastidic SERAT activity (*serat2;1*) or strongly depleted *CYP20-3* expression (*cyp20-3.1* and *cyp20-3.2*), was analyzed with plants that were grown under short-day conditions at a light intensity of 100 μmol m^−2^ s^−1^ until the age of 3 weeks and subsequently challenged with long-day light of 800 μmol m^−2^ s^−1^ irradiance (high light) for additional 10 days. Under control conditions with 100 μmol m^−2^ s^−1^ the growth phenotype of the mutant lines was similar to wild type plants (Figure 2A). Growth under high light conditions caused a decrease in rosette diameter and downward curling of leaves in wild type, *serat2;1, cyp20-3.1*, and *cyp20-3.2* plants when compared to the phenotype of control plants. The mutant genotypes were affected in the same manner as the wild type plants, i.e., also the two *cyp20-3* lines did not differ with respect to phenotypes unlike earlier reports (Cai et al., 2008; Dominguez-Solis et al., 2008). Protein levels of CYP20-3 were neither responding in wild type plants under high light nor in the *serat2;1* mutant. The very low levels of CYP20-3 in *cyp20-3.1* and *cyp20-3.2* plants remained also unchanged (Supplementary Figure 1). Fresh weight determination revealed a 2.2-fold increase in biomass of high light-treated wild type plants compared to control wild type plants (Figure 2B). The fresh weight biomass of *serat2;1, cyp20-3.1*, and *cyp20-3.2* increased only 1.7-, 1.6-, and 2.1-fold, respectively, when grown under high light (Figure 2B). Dry weight measurements of whole rosettes also showed a significant increase of biomass in all genotypes upon high light exposure (Figure 2C). Wild type plants exhibited a 3.5-fold increase, *serat2;1* and *cyp20-3.1* a 2.7-fold increase and *cyp20-3.2* a 4.7-fold increase of dry weight upon high light treatment (Figure 2C).

Along with the stunted growth phenotype red leaf coloring was observed for the high light-treated plants at the abaxial side of the leaves (Supplementary Figure 2). Therefore, anthocyanin levels were determined on the basis of fresh weight (Figure 2D). Significant 2.3-fold to 3.1-fold increases in anthocyanin content could be observed for the wild type, *serat2;1* and both *cyp20-3* mutant lines when compared to anthocyanin levels of unstressed rosette leaves (Figure 2D). Noteworthy, the anthocyanin levels were indistinguishable between wild type and mutant lines, indicating that this stress response is independent of the presence of CYP20-3 or SERAT2;1. Thus, the applied high light conditions had two components: significant stress but also promoted growth. However, in comparison to wild type the *serat2;1* and *cyp20-3.1* and *cyp20-3.2* mutants grew slower under high light conditions. Apparently activation of chloroplast SERAT2;1 by CYP20-3 as well as the presence of SERAT2;1 and thus the chloroplast synthase complex are dispensable, but both systems enhance the performance of Arabidopsis plants under highlight.

### Impact of high light on cysteine metabolism

**Figure 3:**
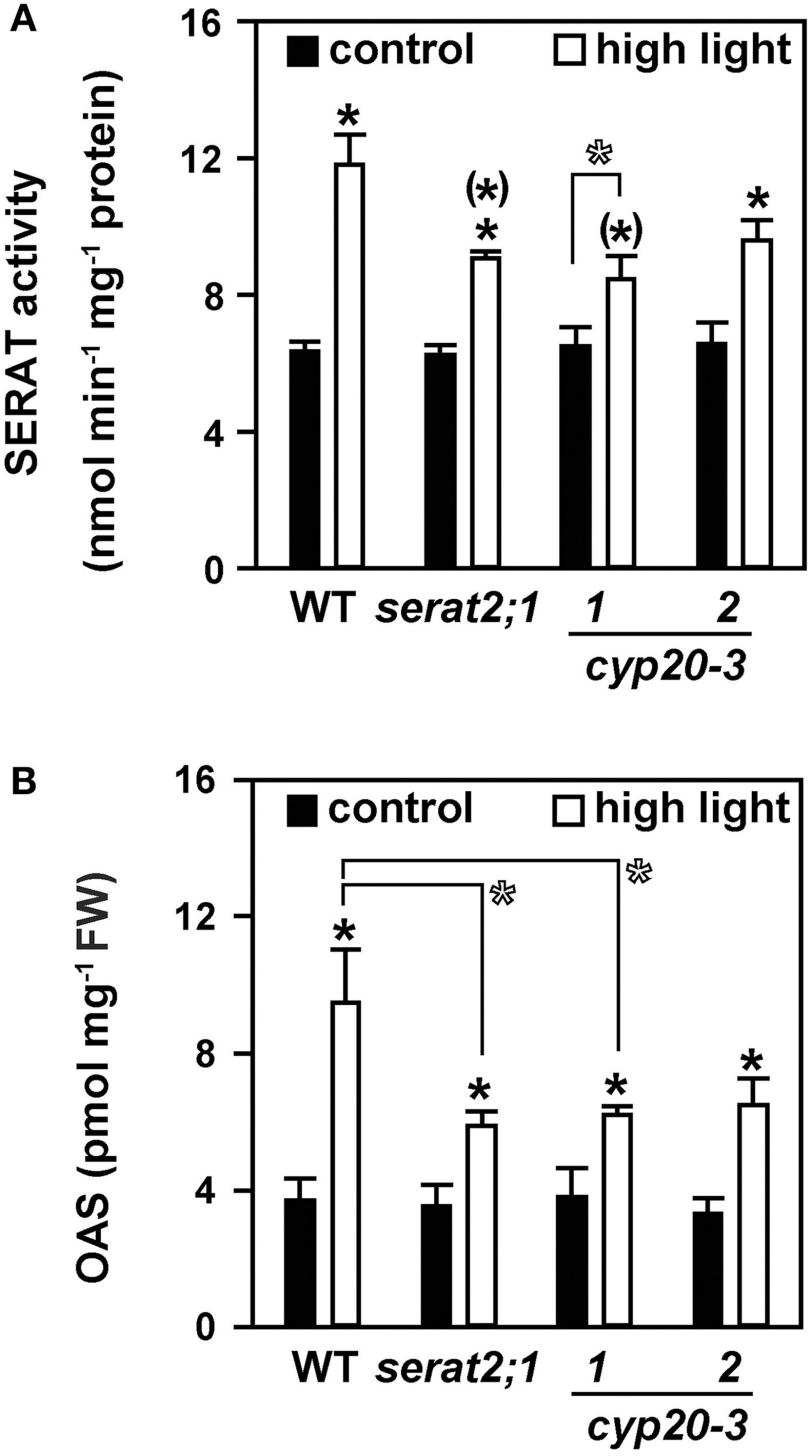
**Effect of 10 days high light treatment on SERAT activity and OAS steady state concentrations in wild type (WT) and *serat2;1, cyp20-3.1*, and *cyp20-3.2* mutant lines**. **(A)** Total SERAT activity was measured in leaf protein extracts of control and high light-treated WT and mutant plants (*n* = 3). **(B)** OAS concentrations were determined from leaf metabolite extracts of control and light-stressed plants (*n* = 5). Black bars indicate control conditions, whereas white bars outline stress conditions. Results represent means ± *SE*. Statistical significances are based on Student's *t*-test. Differences between control and high light treatment are marked with asterisks (*p* ≤ 0.05). Differences between high light treated WT and mutants are marked by asterisks in parentheses (*p* ≤ 0.05). Open asterisks refer to indicated pairwise comparisons with (*p* ≤ 0.1). FW, fresh weight.

To test whether the observed stress susceptibility phenotype of the mutant lines was due to alterations of cysteine metabolism the total SERAT activity and steady-state OAS levels of leaves were determined. Both parameters are established biochemical markers for the state of cysteine synthesis (Haas et al., 2008; Heeg et al., 2008; Watanabe et al., 2008b; Khan et al., 2010). Compared to control conditions a 1.9-fold increase of SERAT activity was observed in high light-treated wild type plants (Figure 3A). A smaller, but still significant increase under high light was also observed for *serat2;1* (1.5-fold) and *cyp20-3.2* (1.5-fold) mutants. SERAT activity was also 1.3-fold elevated in *cyp20-3.1*, but was found at the limit of statistical significance. Higher SERAT activity may result in higher OAS contents. Indeed, changes in OAS concentrations were in agreement with the increased SERAT activity (Figure 3B). A 2.6-fold increase in OAS content was observed for wild type plants exposed to high light conditions. In *serat2;1, cyp20.3.1* and *cyp20-3.2* OAS steady state levels increased 1.7-, 1.6-, and 2-fold, respectively. In accordance with the SERAT activity the raise of OAS concentrations upon high light treatment was smaller in the mutant plants than in the wild type plants. Statistical examination of SERAT activity and OAS content under control conditions revealed no difference between wild type and all three mutant lines. However, the SERAT activities and OAS concentrations in the three mutant lines increased significantly less under high light than those in wild type plants. The weak statistical significance (*p* = 0.106) of the difference of SERAT activity in *cyp20-3.2* to wild type under high light might be owed to the leaky insertion allele and the higher residual CYP20-3 level of this mutant. The same consideration applies to the OAS concentration under high light in *cyp20-3.2* (*p* = 0.120). Thus, plastidic SERAT2;1 and its presumed activation by CYP20-3 is required for fully enhanced OAS synthesis, but about half of the observed increases of SERAT activity and OAS concentrations is likely due to events in mitochondria and cytosol.

**Figure 4:**
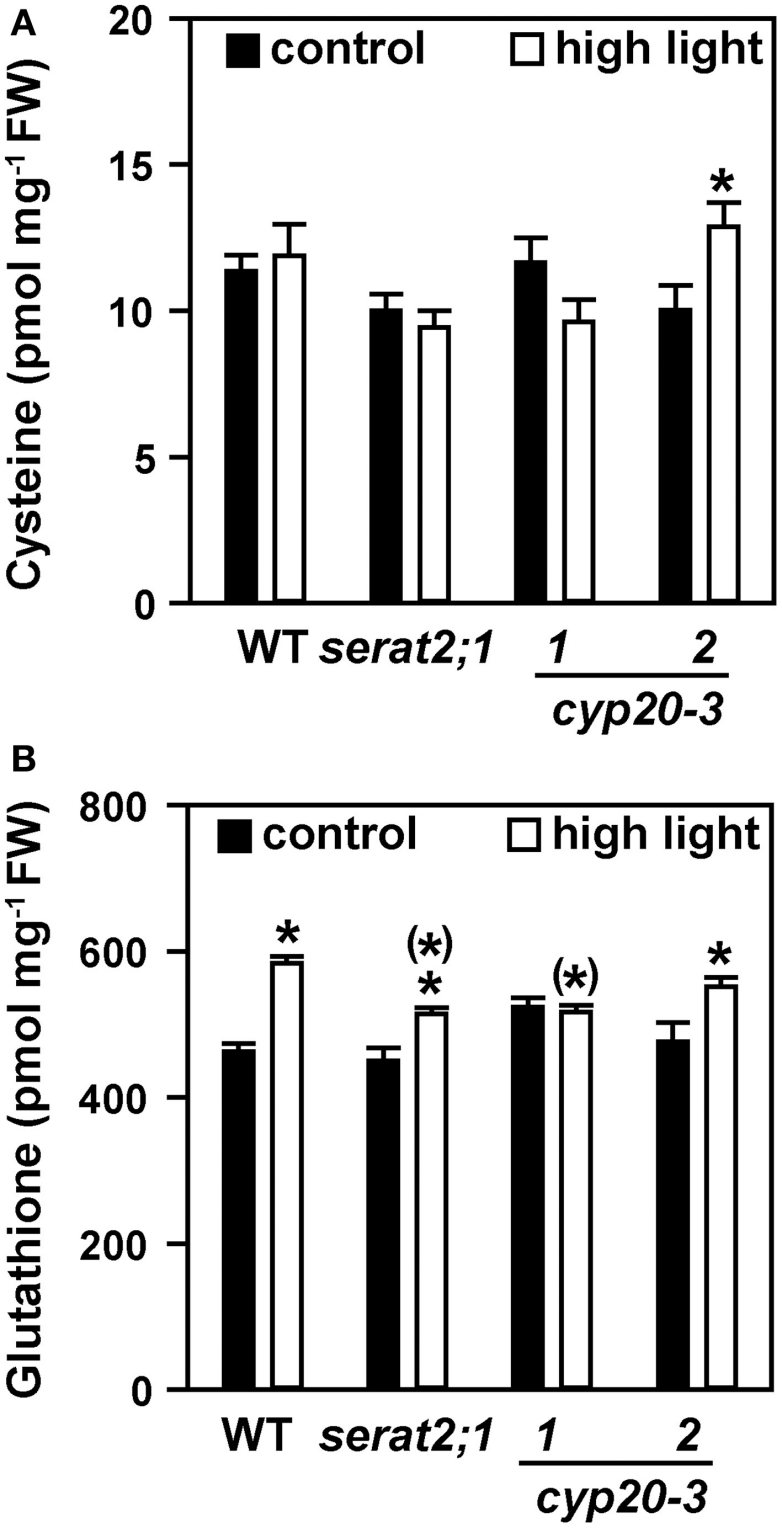
**Effect of 10 days high light exposure on leaf cysteine and glutathione steady state concentrations in wild type (WT) and *serat2;1, cyp20-3.1*, and *cyp20-3.2* mutant lines**. **(A)** Cysteine concentrations were determined from metabolite extracts of control and high light-treated WT and mutant plants. **(B)** Glutathione concentrations. Black bars indicate control conditions, whereas white bars outline stress conditions. Results represent means ± *SE* (*n* = 5). Asterisks indicate statistically significant differences between control and high light treatment (*p* ≤ 0.05; Student's *t*-test). Asterisks in parentheses point to the comparison with high light-treated WT (*p* ≤ 0.05; Student's *t*-test). FW, fresh weight.

Cysteine steady state levels were not significantly altered in high light-exposed wild type, *serat2;1, cyp20-3.1* and *cyp20-3.2* plants compared to unstressed plants (Figure 4A). Glutathione content was moderately but significantly increased in wild type (1.3-fold) upon high light exposure (Figure 4B) similar to previous reports (e.g., Alsharafa et al., 2014). In *serat2;1* (1.1-fold) and *cyp20-3.2* (1.2-fold) the increase was lower than in wild type and found to be unchanged for *cyp20-3.1*. Cysteine and glutathione levels from all three mutant lines were not different from wild type levels under both conditions (Figure 4). Taken together, glutathione steady state levels increase as part of the acclimation response but were similar between wild type and mutants. This steady-state level determination indicates that OAS synthesis is enhanced and, while keeping cysteine levels unchanged, allows for increased glutathione concentrations under high light conditions.

### Impact of high light on SERAT transcript levels

**Figure 5:**
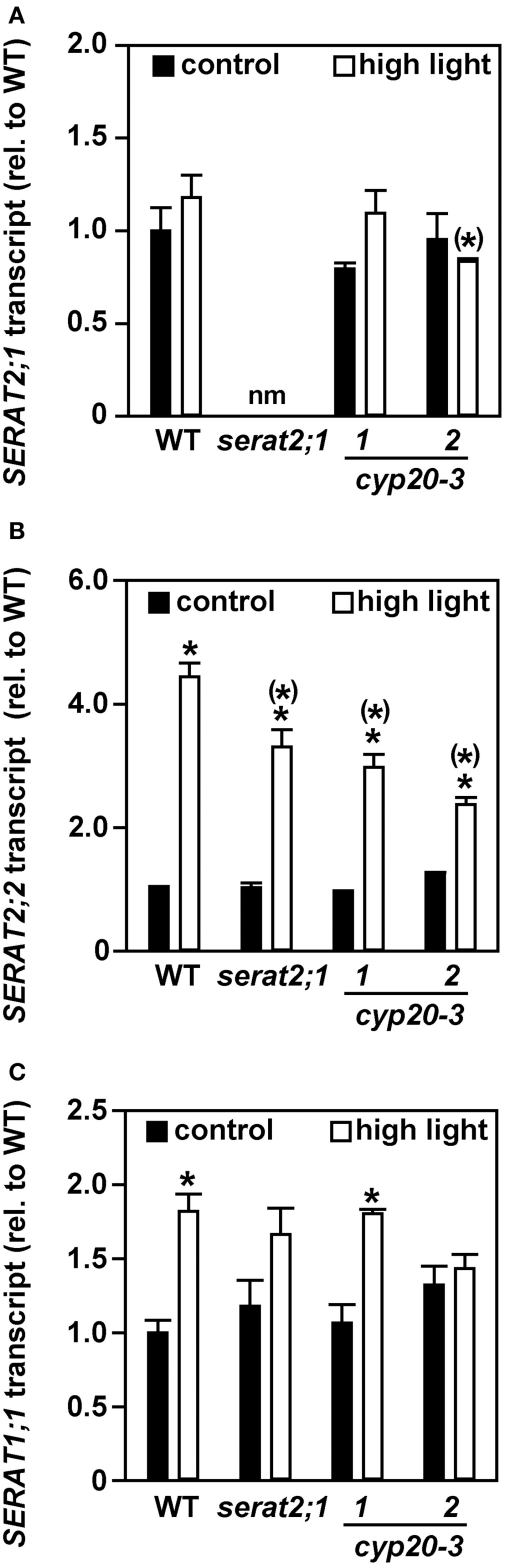
**Effect of high light (10 days) on SERAT transcript levels in leaves of wild type (WT) and *serat2;1, cyp20-3.1*, and *cyp20-3.2* mutant lines**. *SERAT* gene expression levels were determined by qRT-PCR in WT and mutant plants grown under control and high light stress conditions. **(A)** *SERAT2;1* transcripts. **(B)** *SERAT2;2* transcripts. **(C)** *SERAT1;1* transcripts. Black bars indicate control conditions, whereas white bars outline stress conditions. Control WT transcript levels were set to 100%. Results represent means ± *SE* (*n* = 3). Asterisks indicate statistically significant differences between control and high light treatment (*p* ≤ 0.05; Student's *t*-test). Asterisks in parentheses point to the comparison with high light treated WT (*p* ≤ 0.05; Student's *t*-test).

The increase in total SERAT activity and OAS contents upon exposure to high light prompted us to determine the transcript levels of the three major SERAT isoforms. Transcript levels of the plastidic SERAT isoform (*SERAT2;1*) were indistinguishable between control and stressed plants in wild type, *cyp20-3.1* and *cyp20-3.2* lines (Figure 5A). *serat2;1* RNA was not tested in this respect, as this line was already shown to be a full gene knock-out mutant (Watanabe et al., 2008b). A significant increase of the mitochondrial *SERAT2;2* transcript was observed for high light-treated wild type plants (4.4-fold) when compared to wild type control plants (Figure 5B). *SERAT2;2* transcript levels in leaves of *serat2;1* (3.2-fold), *cyp20-3.1* (3.1-fold), and *cyp20-3.2* (1.9-fold) plants were also significantly increased (Figure 5B). Only wild type (1.8-fold) and *cyp20-3.1* (1.7-fold) plants but not *serat2;1* and *cyp20-3.2* lines showed a significant increase in *SERAT1;1* transcript amount (Figure 5C). These expression analyses suggest that mitochondria and possibly to some extent the cytosol provide most of the SERAT activity at least on this intermediate time scale of a 10 day high light treatment for the enhanced synthesis of OAS and ultimately glutathione during high light acclimation to achieve stress mitigation and growth acceleration. The chloroplasts contribute to this process and improve it but are dispensable, at least under the conditions tested.

## Discussion

Earlier analyses of cysteine synthesis in Arabidopsis revealed that under regular growth conditions the chloroplasts provide the substrate sulfide, the mitochondria most of the substrate OAS and the cytosol synthesizes most of the cysteine in leaves, but that all compartments in principle are able to produce cysteine (Haas et al., 2008; Heeg et al., 2008; Watanabe et al., 2008a; Birke et al., 2013). This surprising finding raised the question if there is a specific function of cysteine synthesis in chloroplasts under oxidative stress, since significant upregulation of *SERAT2;1* gene expression was observed after transfer of the CATALASE2 deficient mutant *cat2* from high to ambient CO_2_ concentrations and concomitant H_2_O_2_ stress (Queval et al., 2009) and directly upon H_2_O_2_ exposure (Genevestigator® V3; https://genevestigator.com/gv/plant.jsp). In line with this observation a post-transcriptional upregulation of SERAT2;1 activity by the peptidyl-prolyl *cis/trans*-isomerase CYP20-3 in the chloroplast stroma was linked to resistance against abiotic stress (Dominguez-Solis et al., 2008) and against biotic stress (Park et al., 2013). While a relatively low light intensity which hardly should be considered as stress-inducing for Arabidopsis (300 μmol m^−2^ s^−1^) (Dominguez-Solis et al., 2008) or uncontrolled greenhouse conditions (Cai et al., 2008) had been applied earlier, the controlled acclimation to 800 μmol m^−2^s^−1^ of wild type and mutant plants, either deficient in chloroplast SERAT or strongly depleted of its activation partner CYP20-3 in two mutant alleles, was used as an improved approach to challenge chloroplast cysteine synthesis and indicate a role for this process.

When grown under high light the purple-colored rosette leaves of all genotypes indicated similar anthocyanin accumulation. This confirmed the stress-inducing conditions, since increased anthocyanin levels are considered to reflect stress response to enhanced accumulation of reductive power and carbohydrates (Chalker-Scott, 1999; Vanderauwera et al., 2005; Zeng et al., 2010) and to contribute to scavenging of ROS that are produced in high light-treated plants (Gould et al., 2002). In addition, the glutathione concentration increased in wild type leaves, confirming enhanced demand for cysteine synthesis. This observation was backed up by enriched OAS concentrations, whereas cysteine concentrations remained unchanged in control and high light-treated wild type and mutant plants. This unchanged steady-state might be explained by draining cysteine away to meet the increased demand for glutathione to function as a reductant. This would correspond to the metabolic concept of cysteine as a compound with high turnover but low and rarely changing cellular concentrations (see Hell and Wirtz, 2011, for review). Glutathione levels in turn are known to respond to increasing light intensity but to reach a plateau at 100–200 μmol m^−2^ s^−1^ (Ogawa et al., 2004; Alsharafa et al., 2014). Glutathione was furthermore shown to be essential for the vacuolar sequestration of anthocyanins in maize and petunia through glutathione *S*-transferases (Alfenito et al., 1998; Edwards et al., 2000; Xiang et al., 2001), suggesting that some of the glutathione was removed from the cytoplasmic pool and probably degraded.

However, under the high light conditions employed here (800 μmol m^−2^ s^−1^ for 10 days) the lack of CYP20-3 in the *cyp20-3.1* mutant or the absence of its activation target SERAT2;1 in the chloroplast had only a small effect on the stress phenotypes. Wild type and mutant plants acclimated after transfer to high light, but the increase in fresh and dry weight of mutants lacking SERAT2;1 or CYP20-3 was about 20–30% smaller than that of wild type. Interestingly, the increased total SERAT activities and the resulting OAS concentrations in the four genotypes followed very much the same pattern under high light stress. Thus, the fact that *serat2;1* and *cyp20-3* mutant plants achieve less biomass along with less SERAT activity under high light stress compared to wild type can be attributed to chloroplast SERAT activity. In contrast, Dominguez-Solis et al. (2008) reported an already lowered total SERAT activity in non-stressed *cyp20-3.1* plants and no increase under stress conditions. While it should be cautioned that the treatment conditions and age of plants were different (2 weeks as compared to 4.5 weeks here), the significant increase of total SERAT activity also in the *serat2;1* null mutant demonstrates that the SERAT activities in the other compartments must have responded to the high light treatment.

SERAT2;1 forms the cysteine synthase complex with the OAS-TL B isoform in the stroma of chloroplasts. However, in addition to OAS-TL B the related protein CS26 also consumes OAS: CS26 is a member of the same family of β-substituted alanine synthases like the OAS-TLs and catalyzes the formation of S-sulfocysteine from OAS and thiosulfate in the thylakoid lumen. Thus, CS26 and OAS-TL B potentially compete for the chloroplastidic OAS pool. Intriguingly, expression of the *CS26* gene increases upon transfer to high light according to public databases (Genevestigator® V3; https://genevestigator.com/gv/plant.jsp). Extended transfer of a *CS26* null mutant from short-day to long-day or permanent light (120–160 μmol m^−2^ s^−1^) was reported to result in growth retardation as a result of disturbed redox processes in the thylakoid lumen (Bermúdez et al., 2010, 2012). The control treatment used here is similar to these experiments by shifting *serat2;1* and *cyp20-3* mutants from short-day to long-day. The *serat2;1* mutant was not affected while both *cyp20-3* mutants appeared to grow more slowly, suggesting a possible link to CS26 functions. However, a careful statistical analysis revealed only very weak significance for the growth retardation, at least under the conditions used here. An assessment of photosynthetic parameters of *cyp-20-3* mutants as carried out for the *cs26* mutant (Bermúdez et al., 2012) might be interesting to investigate redox functions of the system. Indeed, the recovery of photosystem II activity was slower in *cyp20-3.1* compared to wild type following photoinhibition by high light treatment (Cai et al., 2008). However, the assumed multiple targets of CYP20-3 reflected by its high abundance (Lippuner et al., 1994; Peltier et al., 2006) makes it difficult to dissect its various functions.

Indeed, expression analysis of the three major *SERAT* genes showed significant increases of *SERAT2;2* mRNA and much less of *SERAT1;1*. The expression of the *SERAT2;1* gene remained unchanged, although regulation could also occur on the transcriptional level: *SERAT2;1* transcript levels were reported to be up-regulated upon treatment of Arabidopsis roots with the oxidizing reagent menadione (Lehmann et al., 2009) and upon oxidative stress in the leaves of the catalase-deficient mutant (*cat2*; Queval et al., 2009). This suggests that the observed increases in total SERAT activity are at least only partially due to activation of SERAT2;1 by CYP20-3 but are complemented by the contributions of the other compartments. In fact, the increase of total SERAT activity in high light-treated wild type plants might be mainly attributed to the induction of *SERAT2;2* transcript amounts in these plants. Several lines of evidence support this assumption. The plastidic SERAT loss-of-function mutant demonstrates that SERAT2;1 makes a small contribution to total SERAT activity in leaves (Watanabe et al., 2008b; Figure 3A). In contrast mitochondrial SERAT2;2 represents approximately 80% of total SERAT activity in Arabidopsis leaves (Watanabe et al., 2008b) and consequently triggers the flux of cysteine synthesis, at least under normal growth conditions (Haas et al., 2008; Wirtz et al., 2012).

A specific contribution of CYP20-3 to the formation of the plastidic cysteine synthase complex by its chaperone function may be unnecessary, since formation of the Arabidopsis cysteine synthase complexes is known to occur spontaneously (ΔG = −33 kcal mol^−1^), without assistance of chaperones (Wirtz et al., 2010) and with an equilibrium dissociation constant of SERAT and OAS-TL subunits of K_D_ = 25 nM (Berkowitz et al., 2002). Given the several hundred-fold excess of OAS-TL over SERAT particularly in chloroplasts of many plant species (reviewed in Hell and Wirtz, 2011), essentially all SERAT molecules can be expected to be bound in the cysteine synthase complex. In addition, measurements of maximal activities of chloroplast SERATs in pea and Arabidopsis indicate only 10–13% of total SERAT activity in chloroplasts (Ruffet et al., 1995; Watanabe et al., 2008b). However, the function of CYP20-3 as a mediator of plastidic CSC formation was reinforced by the demonstration of strongly fostered CYP20-3 and SERAT2;1 interaction in the presence of OPDA (Park et al., 2013). In addition, a sequential pull-down assay showed that OPDA stimulates the interaction between SERAT2;1 and plastidic OAS-TL B with CYP20-3 as the mediator (Park et al., 2013). In line with this function the strong phenotypic and biochemical similarities between *serat2;1* and both *cyp20-3* mutants provide tentative evidence for a joint and specific mechanism. This indicates that, despite having multiple interaction partners, the activation of total SERAT activity upon high light stress is one of the major contributions of CYP20-3 to cope with this stress. The analysis of a mutant lacking chloroplast OAS-TL B protein (*oastlB*; Heeg et al., 2008; Birke et al., 2013) under high light conditions could be suitable to test this hypothesis.

Therefore, the following hypothesis is put forward: CYP20-3 and SERAT1 might contribute to retrograde-signaling of high light stress response instead of direct induction of cysteine synthesis in plastids for enhanced glutathione production. The advantage of this mechanism could be the speed of activation, since OPDA was shown to increase rapidly upon exposure to high light (Alsharafa et al., 2014) which could immediately activate chloroplastidic CSC. This scenario does not exclude that CYP20-3 has also additional functions, which are important under non-stress or other stress condition. Thus, it would not be the plastidic SERAT activity *per se* but the formation of the plastidic CSC itself. CYP20-3 promoted formation of the CSC upon high light might trigger through a yet not characterized signaling cascade the expression of *SERAT2;2* (and probably more) transcripts. The mitochondrial OAS might be transported from mitochondria to plastids and to the cytosol as evidenced (Wirtz et al., 2012; Birke et al., 2013; Lee et al., 2013), to generate cysteine to be used for glutathione biosynthesis. Kinetic analysis of OAS, cysteine and glutathione levels with subcellular resolution would have to be developed to validate this model.

## Materials and methods

### Plant genotypes and growth under control and stress conditions

All experiments were conducted using *Arabidopsis thaliana*, ecotype Columbia-0, as the wild type control and T-DNA insertion mutants, which derived from the same background. Seeds of *cyp20-3.1* (SALK_001615; AT3G62030) and *cyp20-3.2* (SALK_054125; AT3G62030) were obtained from the SALK collection (Salk Institute Genomic Analysis Laboratory) and the *serat2;1* knock-out mutant (SALK_099019; AT1G55920) from Watanabe et al. (2008b). *cyp20-3.2* plants were tested for homozygosity by PCR after Sambrook et al. (1989) with primers 1587_TTTGGCGAAAACTCTTAGCTG), 1588_TGGATTTAACACAAGCGGTTC and 1401_ATTTTGCCGATTTCGGAAC. Genomic leaf DNA was isolated according to Edwards et al. (1991).

Seeds were stratified on soil for 2 days at 4°C and subsequently transferred for germination to growth chambers. Plants were initially grown under short-day conditions with a day/night cycle of 8.5/15.5 h. Humidity was set to 60% and light intensity to 100 μmol m^−2^ s^−1^. After 3 weeks the plants were transferred for 10 days to long-day conditions with a day/night cycle of 16/8 h and light intensities of either 800 μmol m^−2^ s^−1^ (high light) or 100 μmol m^−2^ s^−1^ (low light control). For fresh weight analysis whole rosettes were weighed, for dry weight determination the fresh material was kept for 3–5 days at 60°C beforehand.

### Anthocyanin determination

Determination of anthocyanin content was performed based on the spectral characteristics of cyanidin-derived anthocyanins. The procedure was modified after Giusti and Wrolstad (2001) and Gou et al. (2011). Pigments were extracted in 1 ml 0.1% HCl (in ethanol) from 30 mg ground leaf tissue on a platform shaker at 40 rpm overnight at 4°C. Cell debris was sedimented at 20,000 × g for 10 min at 4°C. Absorbance of the anthocyanin was measured spectrophotometrically at 535 nm and 700 nm and anthocyanin concentration was calculated according to Giusti and Wrolstad (2001).

### Determination of metabolites

Hydrophilic metabolites were extracted from leaves of control and high light-treated Arabidopsis leaves as described in Wirtz and Hell (2003). Thiols and OAS contents were quantified according to Heeg et al. (2008) in cooperation with the Metabolomics Core Technology Platform Heidelberg funded by the DFG Excellence inititative.

### Determination of SERAT enzyme activity and immunological detection of proteins

SERAT activity, coupled to the OAS-TL reaction, was determined based on spectrophotometrical cysteine detection described by Gaitonde (1967). Total soluble protein extracts were prepared from 200 mg ground leaf tissue according to Birke et al. (2013) using Spintrap G-25 columns (GE Healthcare, München). Subsequently protein concentrations as well as enzymatic activities were determined as described by Heeg et al. (2008). Equal amounts of the crude extract were separated by discontinuous SDS-PAGE and blotted on PVDF membrane using a Trans-Blot® Cell system (Bio-Rad, München). Immunological detection of CYP20-3 was done using α-CYP20-3 in combination with a horseradish peroxidase-conjugated secondary antibody and chemiluminescent detection. Loading of protein was tested by staining the gel with Coomassie Brilliant Blue G-250 (Merck, Darmstadt).

### Transcript quantification by qRT-PCR

Gene expression levels were verified in leaves using the principles of the quantification of cDNA targets with quantitative real-time PCR (qRT-PCR). Total RNA was extracted from 100 mg of Arabidopsis leaf tissue using the peqGOLD Total RNA Kit (Peqlab, Erlangen). cDNA was synthesized using the M-MLV Reverse Transcriptase-Kit (Promega, Mannheim). Transcript amount of the respective genes were determined with the Rotor-Gene SYBRGreen PCR Kit (Qiagen, Hilden) and the Rotor-Gene Q system (Qiagen, Hilden).

Gene specific primers used for qRT-PCR: *CYP20-3* (2102_ CTGGACCTGGAATCTTGAGC; 2103_CTTGTCCAAACACGACATGC; 2104_CCACCAAGCATCAGAGAACC; 2105_CCAGCAACTTCACCTCCAAT); *SERAT2;1* (for_CACATGCCGAACCGGTAATAC; rev_GGTGAATCTTCCGGTTTACAGAGA); *SERAT2;2* (for_AATGGAACCCAGACCAAAACC; rev_GCCCAAACATCATCGACTTCA); *SERAT1;1* (for_TGGACACAGATCAAGGCGG; rev_ATGAGAAAGAATCGTCGAATATAGATAGC); *PP2a_PDF2* (for_CTTCTCGCTCCAGTAATGGGACC; rev_GCTTGGTCGACTATCGGAATGCGCG)

### Conflict of interest statement

The authors declare that the research was conducted in the absence of any commercial or financial relationships that could be construed as a potential conflict of interest.
