# Supplementary figures and images for: The significance of cysteine synthesis for acclimation to high light conditions
Source: Front Plant Sci. 2015 Jan 21;5:776. doi: 10.3389/fpls.2014.00776 (PMC4300907; doi:10.3389/fpls.2014.00776)

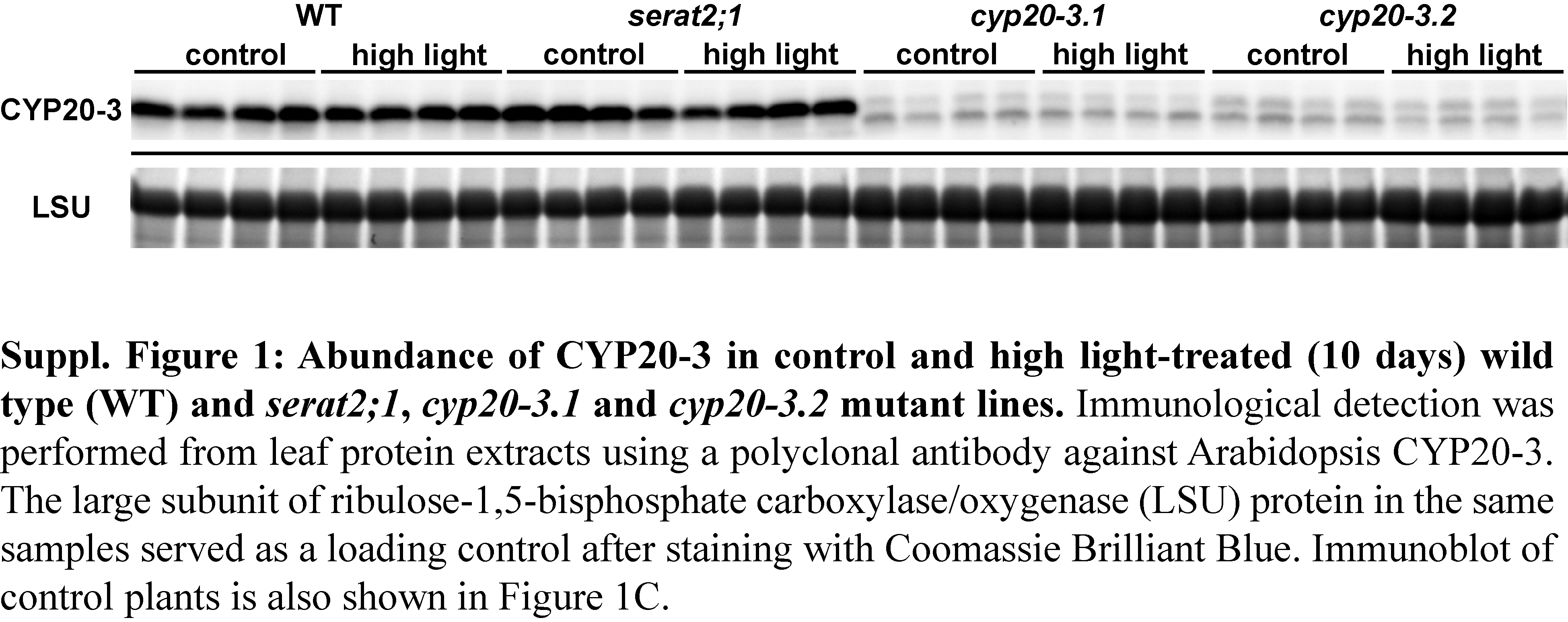

Supplement: Supplementary file 1 [file Image1.TIF]

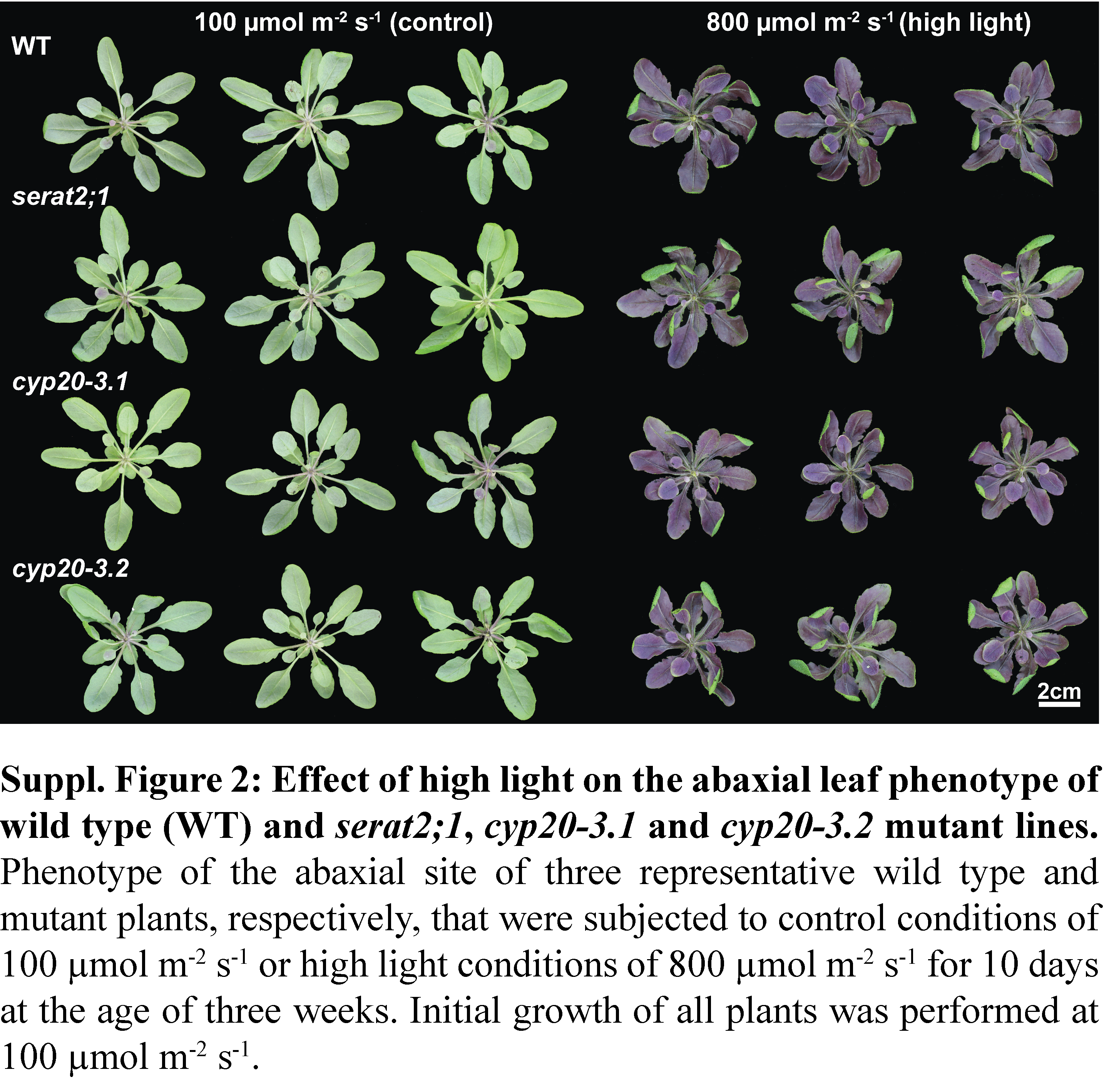

Supplement: Supplementary file 2 [file Image2.TIF]
